# Progress in Gynæcology

**Published:** 1895-09-14

**Authors:** 


### Progress in Gynæcology.

**Electrical Treatment.**—Dr. Lapthorn Smith,[1] of Montreal, advocates very strongly the electrical treatment of fibroids in certain cases. It cannot be used for every case, nor yet by every medical man; nevertheless the positive pole of the galvanic current is in the majority of cases effectual for the cure of pain and bleeding. The plea for the early treatment of fibroids by electricity is quite as just as that for operative treatment, and even more so, for it is entirely devoid of danger, and that can hardly be said of the operative methods. The present position of the electrical treatment is not as good as it ought to be, because it has been tried by men with insufficient experience, and been found wanting by them. In the future, in all probability, electricity will again take its place.

**Dysmenorrhœa.**—The use of the negative pole of the galvanic current for this painful disease, if properly applied, acts in a most marvellous manner. It must also be born in mind, too, that if we cure this disease, we thus get rid of a large number of cases of sterility which are dependent upon it. No less than twenty-three cases which had existed from more than ten years down to over two years are now cured, both of the dysmenorrhœa and sterility, in the last five years. It is not infallible, and will not cure every case, but what other treatment will?

**Removal of Uterine Appendages.**—Dr. Montgomery,[2] of Philadelphia, brings out very strongly the effects of this operation upon the nervous system, the result of prematurely inducing the climacteric which is always a most critical period of a woman's life. The pain is often worse, or as bad, after the operation as before, and the patient becomes very frequently most irritable in temper, very restless, and suffers from insomnia. He winds up by saying that this operation should be done for neuroses only after it has been demonstrated by the experience of capable neurologists that it is the *dernier ressort.*

**Intra-Uterine Medication.**—Landon[3] draws attention to the fact that the danger in this treatment is not perforation of the wall of the uterus, but that it may produce salpingitis, excitation of peristaltic movements and forcing of material into the peritoneum. It is readily forgotten that the colic produced by the introduction of a uterine sound is not only the expression of a uterine contraction, but the tubes also bear a part in the colic. A case is published that occurred in Bonn where a young woman had her uterus curetted for sterility, and afterwards an injection of liq. ferri sesquichlor, and died the same evening. The autopsy showed that the fluid had been forced into the small open veins, a thrombus had formed in the vena cava and caused death. He then lays down the law that injections should not be used for the uterus unless a finger can first be passed in, and thus the exit of the fluid be assured. The best preliminary treatment is dilatation by means of iodoform gauze tampons. Intrauterine manipulations should be restricted as much as possible, injections should not be used at all in an undilated cervix, and the sharp spoon should only be resorted to in extreme cases where exploration indicates disease of the uterine mucous membrane alone.

**Cervical Canal.**—Dr. Herman read a most important paper, in which he has made observations on thirty-four women, as to the dilatation of the cervical canal during menstruation. Statements have before been made on this subject, but they have always been based on theory rather than on experience. By using the largest bougie possible (beginning with one too large) he was able to find out the permeability of the canal. His conclusions are that spontaneous dilatation of the cervical canal, slight in degree, does take place during menstruation; that this dilatation is at its maximum on the third and fourth days of the period; that this dilatation takes place in those who menstruate with pain as well in those who menstruate without, and in those who menstruate scantily as well as in those who menstruate copiously.

**Insufficient Food in Production of Diseases of the Uterus.**—Braithwaite writes somewhat strongly on this point, and brings it out as the missing link in many cases of uterine disease. He thinks that many cases of subinvolution of the uterus are met with in hospital practice which are due to insufficient animal food. It is not always due to the fact that the women are too poor to get the animal food for themselves, but simply they are ignorant of the fact that it is absolutely necessary for them; hence arise many cases of subinvolution not due to or having any bearing on the puerperal period. It may be objected to this theory that there are countries where the food is almost entirely vegetable (such as Egypt) where uterine trouble is almost unknown. This may be explained partly by the fact that in hot countries it is unnecessary to consume much animal food in order to preserve health.

**Varix of the Labium.**—Mr. J. W. Taylor[4] says that in many cases it is highly probable there must be some primary and congenital defect either in the valves of the veins, or in the veins themselves, or in their anastomoses which forms the essential cause of the disease. It is only in this way that an explanation can be offered of the enormous varices met with in quite young persons who are otherwise healthy. We may be inclined to under-estimate the trouble occasioned by these varicose veins, but they are attended by very considerable pain of a dull aching character, and there is always a feeling of danger associated with them. The position forbids the use of a bandage, and other external support is worse than useless. The danger of rupture either externally with free bleeding, or subcutaneously with the formation of a thrombus, makes the condition distressing to the patient and a source of great anxiety to the medical attendant. Minor cases can be treated with more or less advantage by (1) regulation of the bowels and of the menstrual function if the latter is checked or scanty; (2) avoidance of undue exertion or strain; (3) alternation of rest and exercise, and of position. When, however, the veins are always visibly enlarged, tortuous, and prominent, milder measures are useless; and he then goes on to say that some operative treatment is then called for, and he thinks that excision of the varix with antiseptic precautions is far the best.

**Uretero-vaginal Fistulæ.**—Dr. Tuffier,[5] in the last three years, has successfully treated four cases occurring after vaginal hysterectomy. They were all situated on the right side, and this appears to be usually so, and is probably due to the fact that the application of the clamp to the right broad ligament involves on the part of the operator the crossing of his right hand (holding the forceps) over the left hand (which is pulling down the uterus). The ureter may be simply divided, or there may be extensive loss of tissue. In cases where there is loss of tissue, there is usually cicatricial contraction of the uretero-vaginal orifice, and it is very important to remember this, and the operator must be able to overcome the stenosis thus produced, or else there will be no relief given to the affected kidney, for we all know that constriction of the ureter is, sooner or later, followed by destruction of the kidney. It, therefore, follows, as a practical application of this fact, that every operation re-establishing communication between the ureter and bladder should, in the first place, aim at overcoming this stricture, in order not to open into the bladder a canal which will shortly become useless. He thinks that nephrectomy ought never to be performed for the cure of a uretero-vaginal fistula, and it would never be justified except as a last resort. He regards operation through the vagina, for the purpose of leading the ureter into the bladder, as the best. If this fails, he advises that it should be attempted through the abdomen, and, if that cannot be done, then it remains to make the ureter open into the intestine or on to the integument, nephrectomy being the very last resort.

**Dangers of Morphia in Gynæcological Practice.**—Dr. Macnaughton Jones[6] classifies morphia patients under three headings: (1) Those suffering from chronic painful disease who have daily recourse to morphia; (2) those who have been cured of such affections, but continue to use the drug; and (3) those who indulge in morphia for the mere pleasure it affords. Women are more susceptible to the drug than men, reacting readily to its exciting as well as to its sedative action. While it is right to give it for relief of pain, yet it should be withheld in insomnia and the neuroses. Attention was first called to the morphia habit in 1864, and it was about this time that the hypodermic syringe first came into fashion. It is a remarkable fact that a successful means of breaking morphia-takers of their habit was based upon the much greater facility with which it could be abandoned when taken by the mouth. A large proportion of the daily morphia can be cut off without great distress; the crux is reached when the daily quantity is reduced to one or two grains. At this stage the syringe should be abandoned, and even double or treble the quantity given by the mouth. It is then possible to rapidly diminish and at last to altogether abolish the dose without occasioning any very severe distress to the patient. The syringe should really be reserved for cases of great agony requiring immediate relief; a long course of opium, when needed, should be given in other ways; and it should be remembered that it is almost criminal to entrust a patient with a syringe for the self-administration of morphia. On the other hand, in painful and hopeless malignant disease, it is the duty of every conscientious physician not only to alleviate pain where a cure is impossible, but also to ensure a painless death when the end comes. It must not be forgotten that sterility is one of the consequences of morphinism, and instances have occurred in which conception has occurred after the habit has been cured. The risk of using morphia subcutaneously for women suffering from subjective pain and pain arising out of functional disorder of the uterus and ovaries is very great.

**Metrorrhagia.**—In these cases, if dependent upon alterations of the mucous membrane in cases of simple endometritis, M. Labadie-Lagrane[7] has conceived the idea of using a solution of antipyrine and salol. Salol is antiseptic, and liquifies at 109·2 degs. The method is very simple, and the liquid may be prepared at a moment's notice. A test-tube one-third filled with equal parts of salol and antipyrine is heated over a spirit lamp; fusion is produced in two or three minutes, and a slightly brownish liquid is obtained, which, however, has the disadvantage of rapidly becoming solidified when withdrawn from the action of the heat for a few moments, and in order to retard this solidification the tube should be subjected to heat until the mixture becomes of a distinctly brown colour; the product thus obtained will remain liquid for a sufficient length of time. To introduce this liquid into the uterine cavity, a slender, flexible rod is used, at the extremity of which is a small tampon of absorbent cotton saturated with the liquid which has been brought to the proper temperature for the uterus, and this is introduced into the uterine cavity, and the entire surface is painted. This is done two or three times, according to the gravity of the hæmorrhage; then a tampon of cotton saturated with glycerine and creosote is left in the vagina, and the patient is advised to remain in bed. These applications are not at all painful, and have never provoked the least accident. Immediately after the hæmorrhage is arrested, the patient scarcely losing more than a few drops of blood during the day, and afterwards there is no trace of hæmorrhage. It is very rare, he says, to have to make another application after the first.

**Uterine Curetting.**—This operation has not taken the place in this country that it deserves. The chief reason is probably the great importance attached to backward displacement of the uterus, which is generally considered sufficient to account for the menorrhagia and leucorrhœa which a patient may suffer from. Profuse menorrhagia, when not due to fibroids, is most frequently caused by fungous endometritis, and the only remedy for this is to thoroughly curette the uterus. The chief reason that this operation is not more practised is due to the idea that if chronic inflammatory trouble be present in the pelvis or tubes or ovaries this mischief will again be stirred up by any procedure of this kind, but this precaution arose in pre-antiseptic days, before the thorough antiseptic and aseptic treatment of wounds was applied to gynæcology. Before proceeding to the operation the vulva should be cleansed with a solution of corrosive sublimate (1 in 2,000), and the operator's hands, after being very carefully washed and scrubbed, should be rinsed in the same solution; a vaginal injection should then be given, and the instruments should be well soaked in carbolic (1 in 20). The next step is to dilate the cervix, so that the index finger will easily pass into the uterus; this can best be done by Hegar's dilators. While the curette is being passed it should be quite loose in the cervix to ensure delicacy of touch, and the mucous membrane should then be thoroughly scraped down to the muscular coat. The uterus should then be washed out with corrosive sublimate (1 in 4,000), and pure carbolic acid should be applied to the interior of the uterus. This completes the operation. The best time to curette is immediately after the menstrual period, or even on the last day of the catamenia, because the cervix is then more dilatable. If any trouble arises after this operation it is not due to the stirring up of the old mischief, but to the introduction of some septic infection by the operator or his instruments.

[1] N. Y. Med. Record, May 25, 1895. [2] N. Y. Med. Record, May 25, 1895. [3] Therapeutic Gazette, April 15, 1895. [4] Birmingham Medical Review, May, 1895. [5] Medical Week, April 12, 1895. [6] Lancet, April 27, 1895. [7] N. Y. Med. Journal, March 30, 1895. [8] Medical Chronicle, April, 1895.

